# Supplementary material for: Content-rich biological network constructed by mining PubMed abstracts
Source: BMC Bioinformatics. 2004 Oct 8;5:147. doi: 10.1186/1471-2105-5-147 (PMC528731; doi:10.1186/1471-2105-5-147)
Supplement: Additional File 2 — The original results of the above study (non-essential files are deleted to keep the file size under the limit set by BMC bioinformatics). [file 1471-2105-5-147-S2.bz2 › chilibotAdditionalFile2/dip10/399ID9662041E9953/html/EGFR_BTC.html]

 


 **EGFR** and **BTC** 
  
Found 65 abstracts in PubMed, retrieved 10.  
 

 What does Google say? 
 PDF only 
| .edu only 

---

**Interactive relationship** (e.g. stimulation, inhibition, etc)

**Stimulatory relationship**- The results are consistent with a model in which GLP 1 increases PI 3 kinase activity and enhances beta cell proliferation via transactivation of the  **EGFR**  that would require the proteolytic processing of membrane anchored  **BTC**  or other EGF like ligands.  Ref: 12502502 Diabetes, 2003
**Neutral relationship**- Heparin binding epidermal growth factor HB EGF and betacellulin  [ **BTC** ]   **BTC**  are activating ligands for EGF receptor  **EGFR**  ErbB1  [ **EGFR** ]  and ErbB4.  Ref: 12773386 EMBO J< MedlineTA>EMBO J, 2003
- **BTC** , acting through EGF R erbB 1  [ **EGFR** ] , is important for the differentiation of beta cells.  Ref: 12399441 Endocrinology, 2002
- Betacellulin  [ **BTC** ]  and heregulin NDF alpha strikingly colocalized with  **EGFr**  and erbB3 receptors in the granular layer and in a declining gradient from the granular zone to the basal layer, respectively.  Ref: 12768307 Arch Dermatol Res< MedlineTA>Arch Dermatol Res, 2002

**Non-interactive relationship** (e.g. studied together, co-existance, homology, etc.)

- A soluble erbB  [ **EGFR** ]  4 binding fusion protein totally inhibited the effects of NRG 4 but not of  **BTC** .  Ref: 12399441 Endocrinology, 2002
- Pretreatment with effective concentrations of ErbB1  [ **EGFR** ]  inhibitor did not suppress  **BTC**  induced kinase phosphorylation.  Ref: 12475887 FASEB J, 2003
- Moreover, immunohistochemical staining revealed that that  **BTC** , ErbB1  [ **EGFR** ] , and ErbB4 are upregulated in the plaques of human atherosclerotic coronary arteries.  Ref: 12869389 Circ ResCirc Res, 2003
- **BTC**  induced phosphorylation of all four EGF receptors present on HTASMCs ErbB1  [ **EGFR** ] , ErbB2, ErbB3, and ErbB4.  Ref: 12869389 Circ ResCirc Res, 2003
- Reverse transcription polymerase chain reaction was performed on each sample with primers for rat epidermal growth factor, epidermal growth factor receptor  [ **EGFR** ]  ErbB  [ **EGFR** ] , heparin binding epidermal like growth factor, hepatocyte growth factor, hepatocyte growth factor receptor, keratinocyte growth factor, betacellulin  [ **BTC** ] , amphiregulin, and neuregulin alpha.  Ref: 12148846 Laryngoscope, 2002
- Both the c Src inhibitor PP1 and the  **EGFR**  specific inhibitor AG1478 blocked GLP 1 induced 3 H thymidine incorporation in INS 832 13 cells as well as in isolated rat islets, while only AG1478 inhibited the proliferative action of betacellulin  [ **BTC** ]   **BTC** , an  **EGFR**  agonist.  Ref: 12502502 Diabetes, 2003
